# Supplementary material for: Oropouche virus outbreaks in northeast Brazil between 2024–25 are characterized by sustained transmission and spread to newly affected areas
Source: PLoS Negl Trop Dis. 2026 Apr 1;20(4):e0014171. doi: 10.1371/journal.pntd.0014171 (PMC13056256; doi:10.1371/journal.pntd.0014171)
Supplement: S1 File — Municipal Patterns of Oropouche Fever Incidence in Northeastern Brazil. (DOCX) [file pntd.0014171.s001.docx]

**Supplementary Results for the study**

Oropouche virus outbreaks in northeast Brazil between 2024-25 are characterized by sustained transmission and spread to newly affected areas

Elverson Soares de Melo ^a,b *^, Sophia Maria Dantas da Silva ^c^, Gustavo Barbosa de Lima ^d^, Adalúcia da Silva ^d^, Alexandre Freitas da Silva ^a^, Verônica Gomes da Silva ^d^, Elisa de Almeida Neves Azevedo ^d^, Letícia Welter Rother ^e^, Keilla Maria Paz e Silva ^f^, Diego Arruda Falcão ^f^, Andreza Pâmela Vasconcelos ^f^, Mayara Matias de Oliveira Marques da Costa ^f^, Eduardo Augusto Duque Bezerra ^g^, Thiago Franco de Oliveira Carneiro ^h^, Erik Matthaus de Lima Paiva ^h^, Janaina Correia Oliveira ^h^, Matheus Filgueira Bezerra ^i^, Marcelo Henrique Santos Paiva ^a^, Bartolomeu Acioli-Santos ^d^, Clarice Neuenschwander Lins de Morais ^d^, Tulio de Lima Campos ^j^, Gabriel da Luz Wallau ^a, k, l *^

^a^ Department of Entomology and Bioinformatics Core, Aggeu Magalhães Institute (IAM), Oswaldo Cruz Foundation (Fiocruz/PE), Recife, Brazil

^b^ Federal University of Pernambuco (UFPE), Recife, Brazil

^c^ Graduate Program in Public Health, Aggeu Magalhães Institute (IAM), Oswaldo Cruz Foundation (Fiocruz/PE), Recife, Brazil

^d^ Department of Virology, Aggeu Magalhães Institute (IAM), Oswaldo Cruz Foundation (Fiocruz/PE), Recife, Brazil

^e^ Graduate Program in Agronomy, Federal University of Santa Maria (UFSM), Santa Maria, Brazil

^f^ Central Laboratory of Public Health of Pernambuco (LACEN-PE), Recife, Brazil

^g^ Pernambuco State Department of Health, Recife, Brazil

^h^ Central Laboratory of Public Health of Paraíba (LACEN-PB), João Pessoa, Brazil

^i^ Department of Microbiology, Aggeu Magalhães Institute (IAM), Oswaldo Cruz Foundation (Fiocruz/PE), Recife, Brazil

^j^ Bioinformatics Core, Aggeu Magalhães Institute (IAM), Oswaldo Cruz Foundation (Fiocruz/PE), Recife, Brazil

^k^ Department of Arbovirology and Entomology, Bernhard Nocht Institute for Tropical Medicine, WHO Collaborating Center for Arbovirus and Hemorrhagic Fever Reference and Research, National Reference Center for Tropical Infectious Diseases, Hamburg, Germany

^l^ Federal University of Santa Maria (UFSM), Santa Maria, Brazil

* Corresponding Authors: Melo ES (elverson.melo@gmail.com), Wallau GL (gabriel.wallau@fiocruz.br)

**Supplementary results**

***Municipal Patterns of Oropouche Fever Incidence in Northeastern Brazil***

Although Maranhão was the first state to report autochthonous transmission of Oropouche fever, it had the lowest incidence in the region, with 0.52 cases per 100,000 inhabitants. Cases occurred across all its Intermediate Regions (IR), with the city of Cidelândia recording the highest number of cases (6) and the highest municipal incidence (45.6 per 100,000). In neighboring Piauí, the lowest proportion of affected municipalities (2.77%) was observed, along with the second-lowest state-level incidence. Most cases were concentrated in Amarante (16 cases), and the highest municipal incidence occurred in Jardim do Mulato (94.1 per 100,000), both located in the IR of Teresina near the Maranhão border. In Bahia, which reported the highest number of cases in 2024, the most affected municipalities were Ilhéus (138 cases), Gandu (82), and Uruçuca (73), with the latter one having the highest municipal incidence (329.87 per 100,000). Most high-incidence municipalities were located south of Salvador, within the IR of Santo Antônio de Jesus and Ilhéus-Itabuna. In Alagoas, approximately 80% of cases were reported in Palmeira dos Índios, the municipality with the highest incidence, situated inland near the Pernambuco border. In Sergipe, a similar pattern was observed, with most cases concentrated in Siriri, which reported 18 cases and the highest municipal incidence in the state. In Ceará, unlike Alagoas and Sergipe, cases were more geographically dispersed among neighboring municipalities, particularly in the northern part of the state. The municipalities of Aratuba, Baturité, Capistrano, Pacoti, and Mulungu recorded the highest case counts and incidences (**Fig. 3B**).

In Pernambuco, although municipal-level Oropouche fever coverage was the highest among states in the region, transmission exhibited substantial local heterogeneity. In 2024, autochthonous cases were reported in 28 municipalities, the majority of which were located in the eastern part of the state, within the Recife Intermediate Region (**Fig. 3A**). Jaqueira (68 cases; 648.6 per 100,000 inhabitants) and Timbaúba (12 cases; 25.2 per 100,000 inhabitants) together accounted for more than half of the total cases reported. Additionally, 45% of the affected municipalities recorded only a single confirmed case, reflecting the marked spatial disparity in case distribution across the state. In 2025, Goiana, a municipality located in the northern part of Pernambuco near the border with Paraíba, was the only location with confirmed local transmission up to May, suggesting limited but persistent virus circulation into the following year. Paraíba, the last state in Northeastern Brazil to report autochthonous transmission of Oropouche fever, experienced a sharp increase in cases during early 2025. In 2024, only six locally acquired cases were reported, occurring in three municipalities: Alagoa Nova (3 cases), Campina Grande (2 cases), and João Pessoa (1 case). By May 2025, however, the number of confirmed cases had grown to 664, with 17 municipalities reporting transmission, mainly in interior regions of the state. The highest burden occurred in Bananeiras, which reported both the largest number of cases and the highest incidence rate in the Northeast (1,800.8 cases per 100,000 inhabitants). Several other municipalities also reported high incidence rates compared to the regional average, including Matinhas (35 cases; 739.1 per 100,000), Alagoa Nova (39 cases; 179.5 per 100,000), Massaranduba (18 cases; 122.7 per 100,000), Lagoa Seca (27 cases; 92.9 per 100,000), Alagoa Grande (18 cases; 67.2 per 100,000), and Campina Grande (32 cases; 7.25 per 100,000).

***Moran and LISA analysis***

The four clusters of high disease incidence are located in southern Bahia (25 municipalities), Ceará (9 municipalities within the Fortaleza Health Region), Paraíba (8 municipalities within the Campina Grande Health Region), and the southern forest zone of Pernambuco (2 municipalities) (**Fig. S4**). In addition to these clusters, 27 municipalities were classified as Low-High spatial outliers, suggesting transitional areas with potential for transmission expansion or regions with possible case underreporting. These municipalities were mainly concentrated in five geographic areas: northern Ceará (Caridade, Canindé, Itapiúna, Aracoiaba, Ibaretama, and Acarape), southern Bahia (Nilo Peçanha, Brejões, Jiquiriçá, Varzedo, Santa Terezinha, Jaguaripe, and Buerarema), southern Pernambuco (Lagoa dos Gatos and São Benedito do Sul), Paraíba (Esperança, São Sebastião de Lagoa de Roça, Serra Redonda, Pirituba, Belém, Dona Inês, and Tacima), and Alagoas (Mar Vermelho and Belém, both neighboring Palmeiras dos Índios). Nevertheless, most municipalities in the Northeast region exhibited low incidence rates and no significant spatial clustering, suggesting a limited geographic spread of the disease.
