# Supplementary figures and images for: Oropouche virus outbreaks in northeast Brazil between 2024–25 are characterized by sustained transmission and spread to newly affected areas
Source: PLoS Negl Trop Dis. 2026 Apr 1;20(4):e0014171. doi: 10.1371/journal.pntd.0014171 (PMC13056256; doi:10.1371/journal.pntd.0014171)

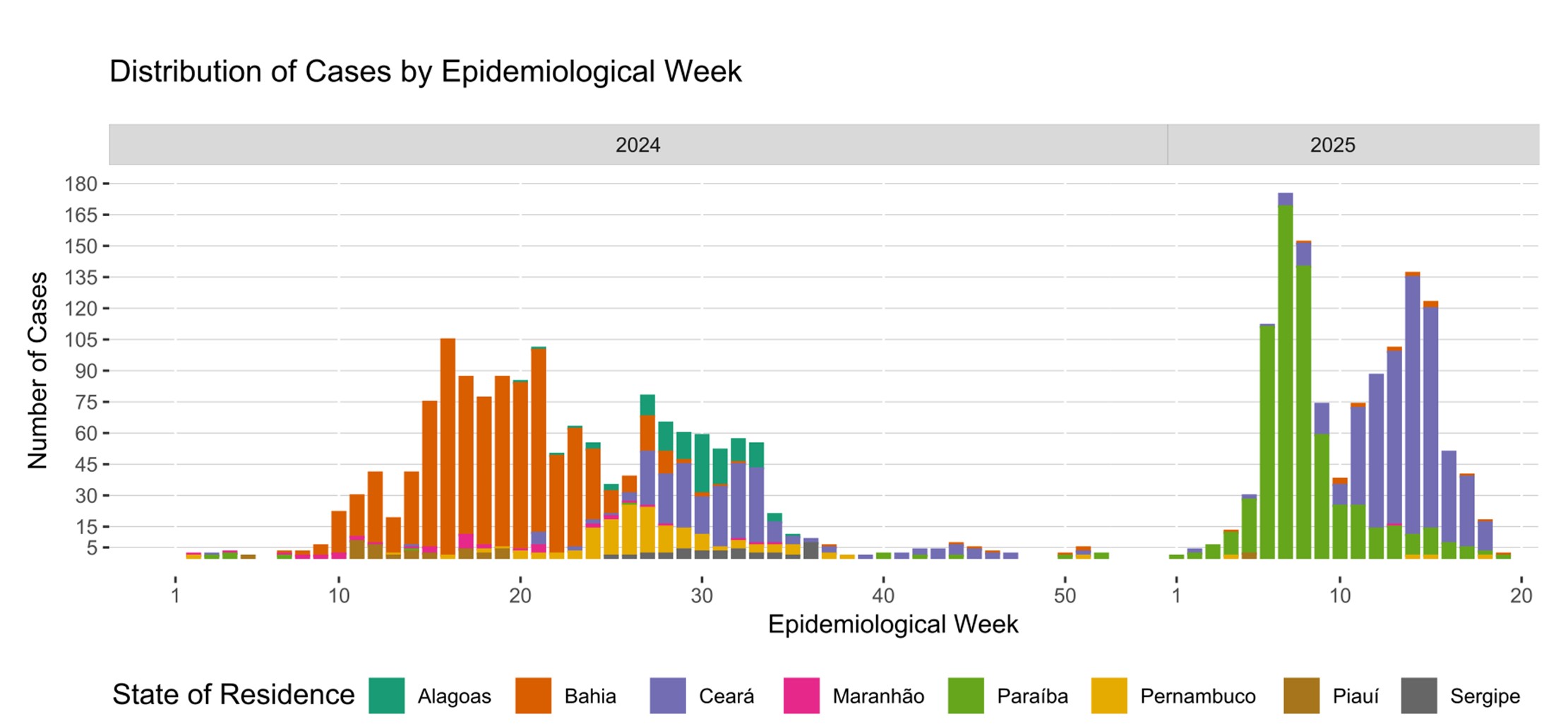

Supplement: S1 Fig — (JPG) [file pntd.0014171.s003.jpg]

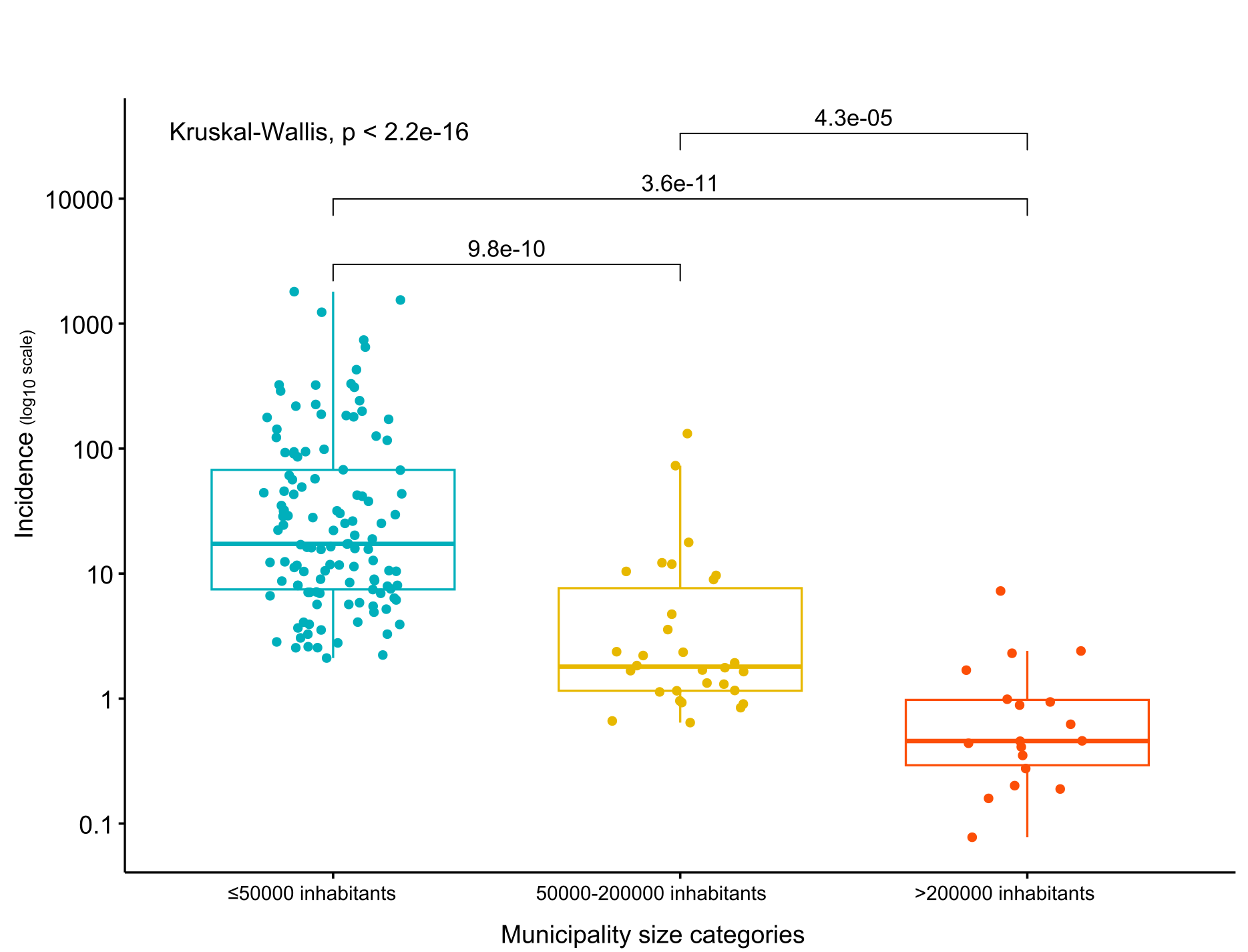

Supplement: S2 Fig — Each point represents a municipality. Boxplots show the median and the interquartile range (IQR). Differences between groups were tested using the Kruskal–Wallis test (p < 2.2 × 10 ⁻ ¹⁶). Brackets indicate all pairwise comparisons, with their p-values. Numeric values are shown on the x-axis, but the axis is on a logarithmic scale. (PNG) [file pntd.0014171.s004.png]

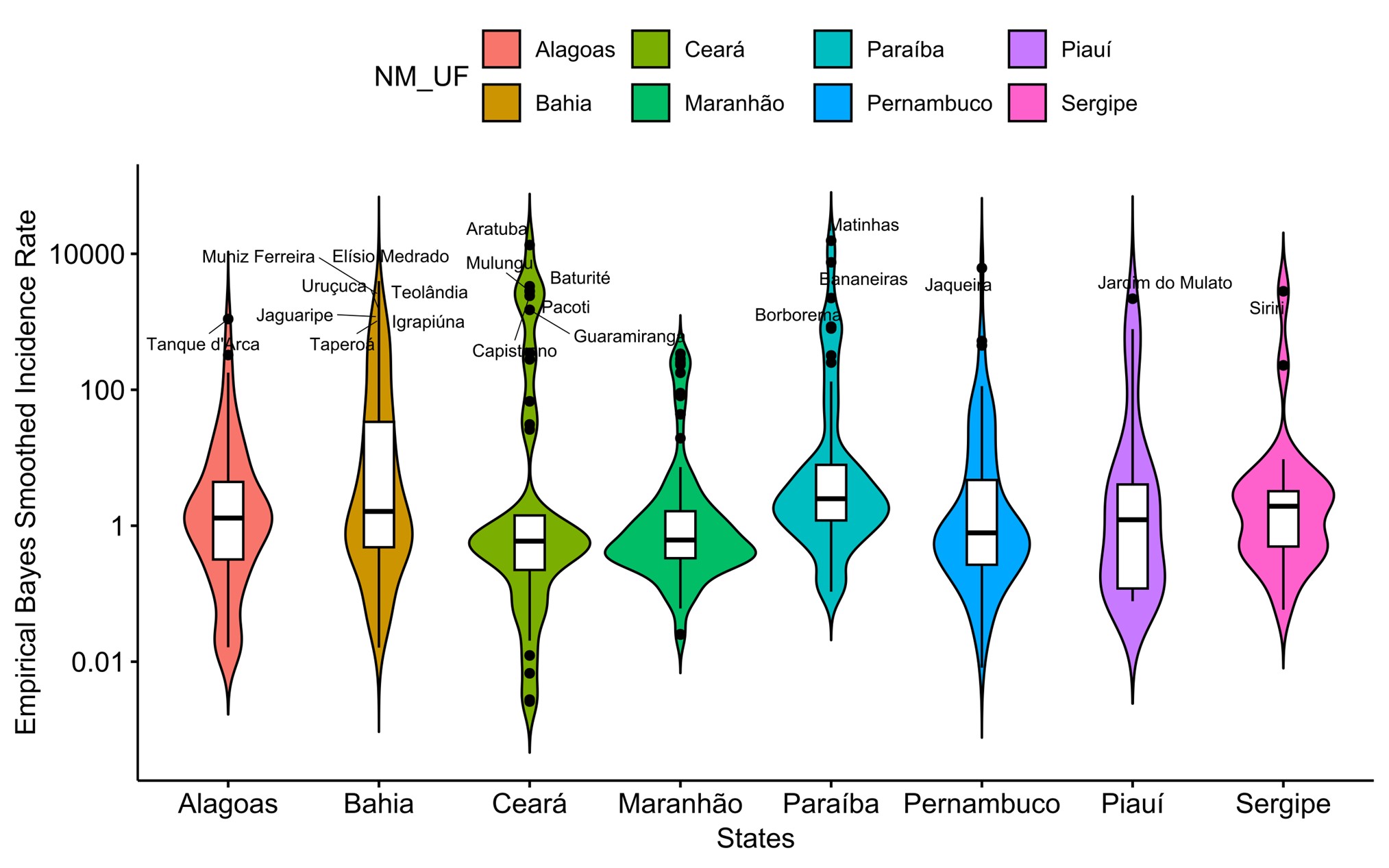

Supplement: S3 Fig — Each violin plot represents the distribution across municipalities whose smoothed incidence is greater than zero. The names of municipalities with smoothed incidence higher than 1000 are highlighted. (JPG) [file pntd.0014171.s005.jpg]

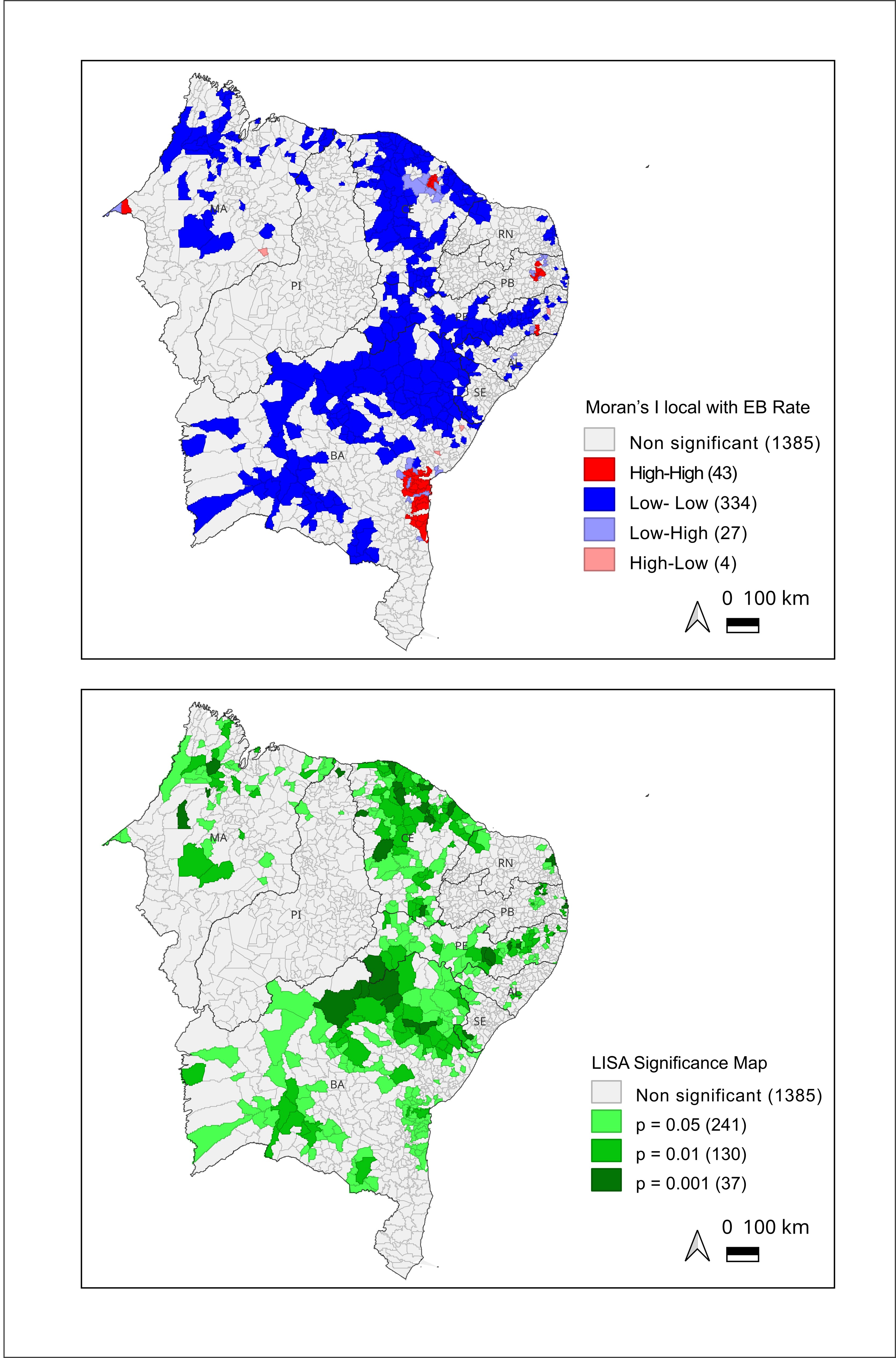

Supplement: S4 Fig — Top panel: Cluster map showing spatial autocorrelation patterns. Bottom panel: LISA significance map indicating municipalities with statistically significant local spatial autocorrelation at p < 0.05, 0.01, and 0.001. The basemap shapefiles used to produce this figure were obtained from the IBGE municipal mesh dataset (available at: https://geoftp.ibge.gov.br/organizacao_do_territorio/malhas_territoriais/malhas_municipais/municipio_2023/Brasil/BR_Municipios_2023.zip), which is distributed under a CC BY 4.0 license (https://biblioteca.ibge.gov.br/visualizacao/livros/liv102169.pdf) (JPG) [file pntd.0014171.s006.jpg]

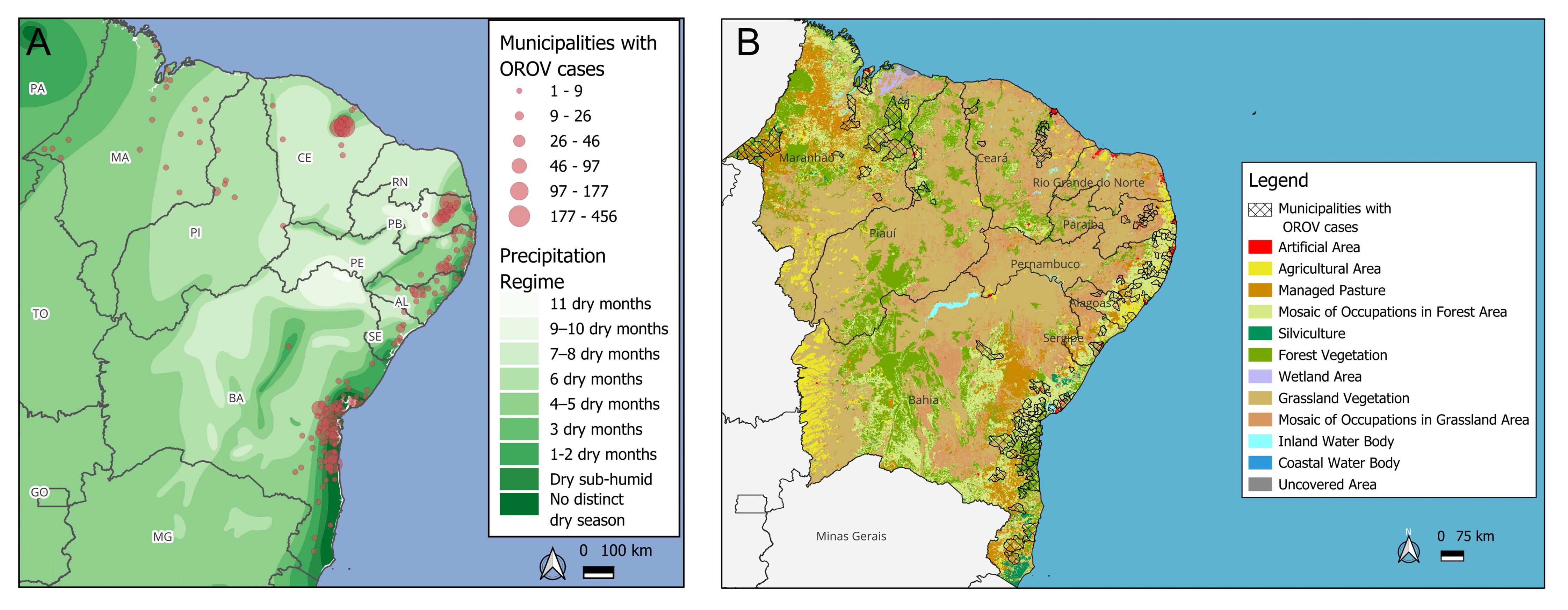

Supplement: S5 Fig — The basemap shapefiles used to produce this figure were obtained from the IBGE climate dataset (available at: http://geoftp.ibge.gov.br/informacoes_ambientais/climatologia/vetores/brasil/Clima_5000mil.zip) and the IBGE land cover and use dataset (available at: https://www.ibge.gov.br/geociencias/downloads-geociencias.html?caminho=informacoes_ambientais/cobertura_e_uso_da_terra/monitoramento/grade_estatistica/serie_revisada_2022/vetores_compactados/UFs, both distributed under a CC BY 4.0 license (https://biblioteca.ibge.gov.br/visualizacao/livros/liv102169.pdf) (JPG) [file pntd.0014171.s007.jpg]

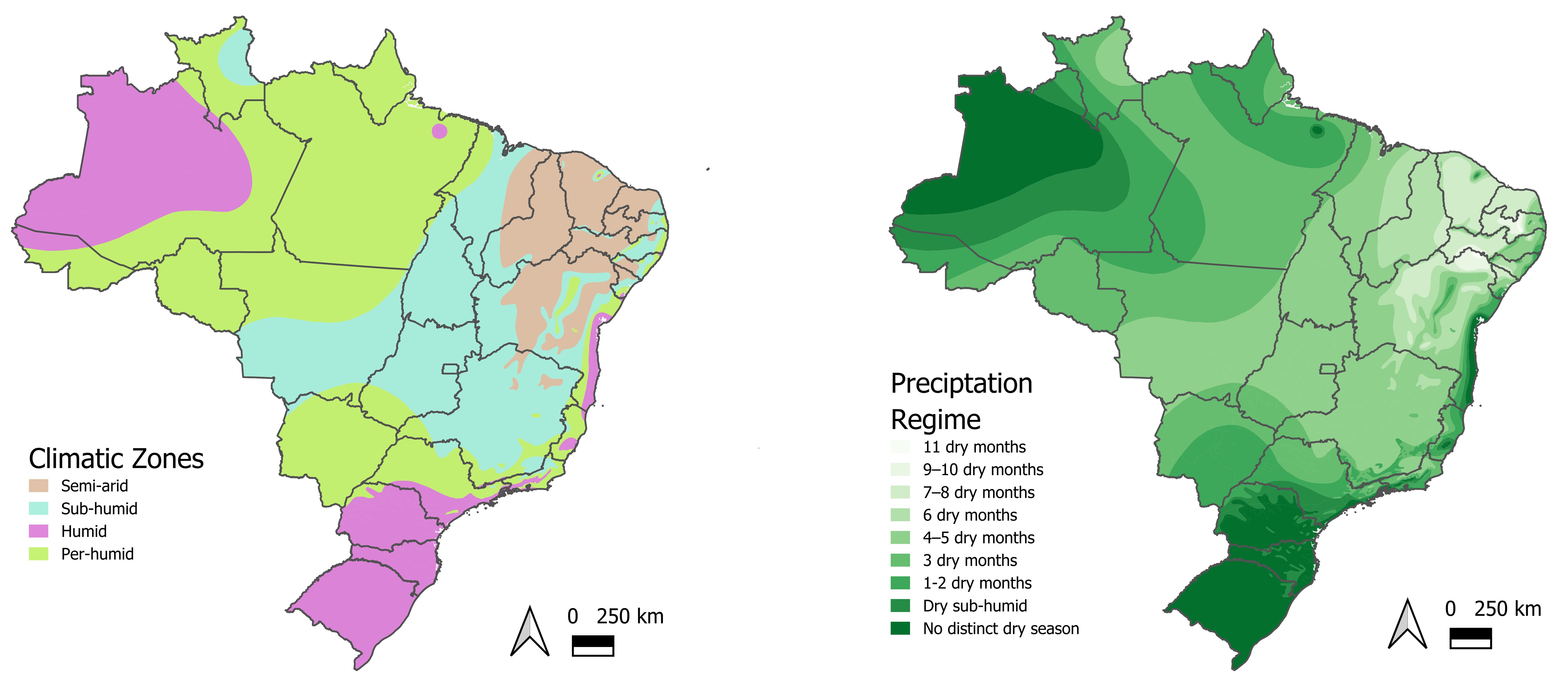

Supplement: S6 Fig — The basemap shapefiles used to produce this figure were obtained from IBGE climate dataset (available at: http://geoftp.ibge.gov.br/informacoes_ambientais/climatologia/vetores/brasil/Clima_5000mil.zip), which is distributed under a CC BY 4.0 license (https://biblioteca.ibge.gov.br/visualizacao/livros/liv102169.pdf) (JPG) [file pntd.0014171.s008.jpg]

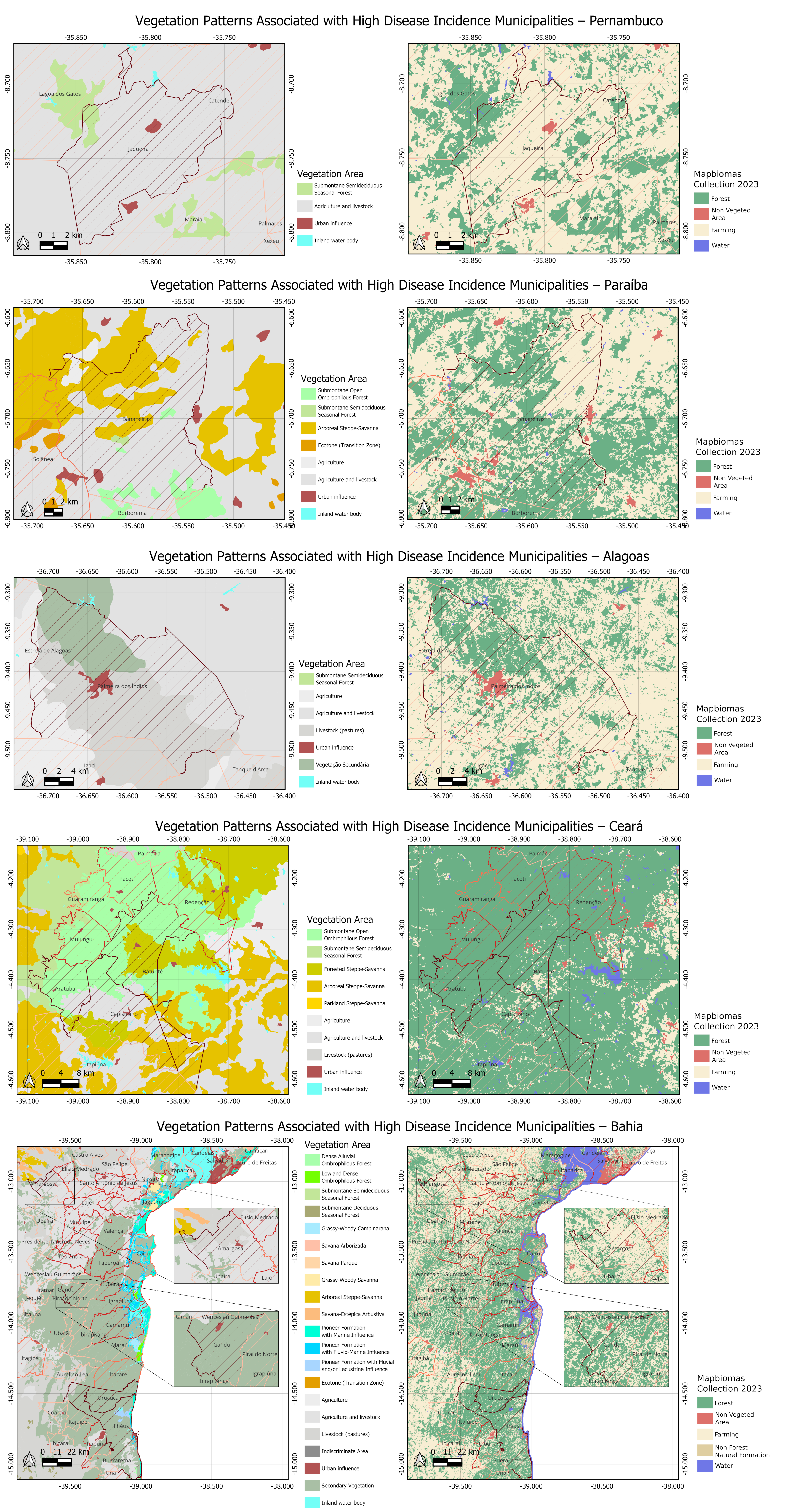

Supplement: S7 Fig — Each panel compares vegetation cover (left) and land use classification from MapBiomas 2023 (right), highlighting the presence of forest fragments, transitional zones, and agricultural areas within affected municipalities. The basemap shapefiles used to produce this figure were obtained from the IBGE Brazilian vegetation dataset (available at: https://geoftp.ibge.gov.br/informacoes_ambientais/vegetacao/vetores/escala_250_mil/versao_2023/) and the MapBiomas Collection 2023, which provides an annual series of land cover and land use maps of Brazil (accessed via the official MapBiomas Collection plugin for QGIS: https://github.com/mariochermes/mapbiomascollection), distributed under a CC BY 4.0 license (https://brasil.mapbiomas.org/en/termos-de-uso/). (PNG) [file pntd.0014171.s009.png]

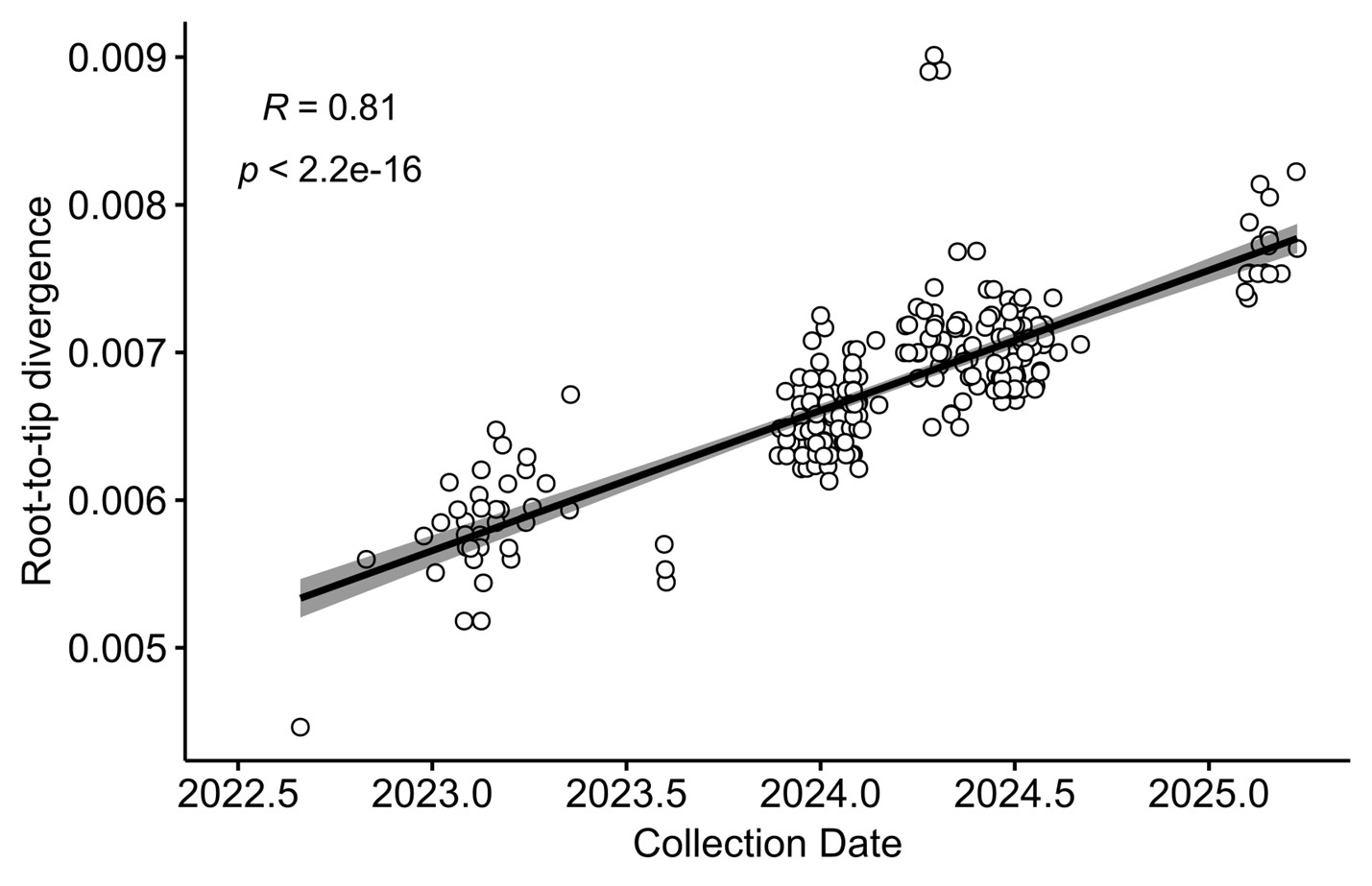

Supplement: S8 Fig — (JPG) [file pntd.0014171.s010.jpg]
