# Supplementary material for: Oropouche virus outbreaks in northeast Brazil between 2024–25 are characterized by sustained transmission and spread to newly affected areas
Source: PLoS Negl Trop Dis. 2026 Apr 1;20(4):e0014171. doi: 10.1371/journal.pntd.0014171 (PMC13056256; doi:10.1371/journal.pntd.0014171)
Supplement: Attachment — Submitted filename: PNTD-D-25-01580 Review comment.docx [file pntd.0014171.s011.docx]

PNTD-D-25-01580

It is my honor to review this research article about the Oropouche disease outbreak in Northeast Brazil between 2024-25. The authors provide a comprehensive analysis utilizing the epidemiological and molecular data. Here are my comments:

**Introduction**

In the 4^th^ paragraph of the Introduction, I suggest to update the number of confirmed OROV cases in other American countries using the most recent Pan American Health Organization (PAHO) Epidemiological Update Oropouche in Americas Region on August 13, 2025 (<https://www.paho.org/en/documents/epidemiological-update-oropouche-region-americas-13-august-2025>).

I suggest adding a sentence in the Introduction to briefly introduce the Northeast Region in Brazil. For example, how many states it includes, which states they are, and that is the second most populus region in Brazil. This information would help readers unfamiliar with Brazil better understand the geographic and demographic context of this research. Since there are five paragraphs in the Introduction, the authors can decide the most appropriate location for inserting this information.

**Materials and methods**

Under the section “Segment-Based Phylogenetics and Recombination Filtering,” if Lima et al. (2025) refers to the same paper cited as reference 15, please update the citation to Lima et al. (2024).

**Results**

In the Result section, I noticed that epidemiological week (EW) is the main time unit used to describe confirmed OROV cases. To improve clarity and consistency, I suggest spelling out “epidemiological week (EW)” the first time it is mentioned, and then using only EW in all subsequent references. In addition, I recommend including the actual dates in parentheses. For example, EW 10 in 2024 (March 3-9) instead of using vague terms like “early March,” so that readers have a more precise understanding of the timeline. This suggestion applies to all similar instances throughout the Results section.

Under the section “Epidemiological Dynamics of Oropouche Fever in the Northeast of Brazil”, I suggest using “indicating continuous spreading” instead of “indicating an acceleration in the spread of the virus.” While it is true that 11,300 cases were confirmed from January to May 2025, compared with 13,800 confirmed cases in all of 2024, this period corresponds to the wet season when most cases are typically expected. Therefore, there may not be sufficient evidence to conclude an acceleration in virus spread. Thus, I believe describing it as continuous spread is more appropriate.

Under the section “Epidemiological Dynamics of Oropouche Fever in the Northeast of Brazil,” the phrase “(~xx% of national cases)” is used to describe the spread of OROV in the Northeast region. I suggest adding a time frame to provide more precise context. For example, “1517 cases of Oropouche fever in 2024 (~11% of 2024 national cases).” The same approach should be applied for 2025: “(~11.4% of 2025 national cases).”

Under the section “Epidemiological Dynamics of Oropouche Fever in the Northeast of Brazil,” the beginnings of the 2^nd^ and 3^rd^ paragraphs provide different information, which may disrupt the flow. In 2^nd^ paragraph: “Autochthonous transmission in Northeast Brazil was first documented in **March**…” In 3^rd^ paragraph: “Although the first autochthonous cases were officially reported in **February** 2024…” Please double check.

Under the section “Epidemiological Dynamics of Oropouche Fever in the Northeast of Brazil,” could the author provide the number of municipalities in Ceará state that reported confirmed cases in 2024 and again in 2025, particularly where case numbers increased? However, I think this may reflect a continuation of the outbreak from late 2024 into the first half of 2025 as the wet season continuous.

**Table 1**. I suggest adding a column showing the number of the municipalities in each state as these numbers vary widely: from 75 in Sergipe to 417 in Bahia. Reporting only the percentage does not provide readers with a complete understanding the municipal coverage.

Under the section “Municipal Patterns of Oropouche Fever Incidence in Northeastern Brazil”, in the 3^rd^ paragraph, I understand Campina Grande is a municipality in Paraíba but what does “Campina Gande IR” refer to? Cloud this be a typo?

**Figure 2**. I may have missed it, but how do the authors distinguish between autochthonous and improved OROV cases?

**Discussion**

In addition to the fact that “Oropouche fever has historically been underreported in many parts of Brazil and presents symptoms that resemble those other arboviral infections,” another reason is that asymptomatic Oropouche infections are not captured by lab-confirmed cases. Therefore, one important future study recommendation would be to conduct population-based serological surveys to access the true seroprevalence in a specific location. While the authors suggest randomized serological surveys, it is not clear how this approach would capture the population most affected. Conduct population-based serosurveys in locations both with and without reported lab-confirmed cases would provide a more comprehensive understanding, allowing estimations of seroprevalence before and after an outbreak and calculation of the attack rate during an outbreak.

Thank you.
