# Supplementary material for: Oropouche virus outbreaks in northeast Brazil between 2024–25 are characterized by sustained transmission and spread to newly affected areas
Source: PLoS Negl Trop Dis. 2026 Apr 1;20(4):e0014171. doi: 10.1371/journal.pntd.0014171 (PMC13056256; doi:10.1371/journal.pntd.0014171)
Supplement: Attachment — Submitted filename: Response to Reviewers.docx [file pntd.0014171.s013.docx]

Dear Editor,

We thank you and the reviewers for the careful evaluation of our manuscript. We appreciate the constructive feedback and have revised the manuscript accordingly. In addition to addressing the reviewers’ comments, we have also complied with all journal technical requirements, including completing the CRediT author contributions, adding an Author Summary, uploading all main figures as separate TIFF files, providing Supporting Information legends, clarifying map data sources and licensing for all figures, and revising the Financial Disclosure and Competing Interest statements per PLOS guidelines. Below, we provide a point-by-point response to each comment. All revisions have been incorporated into the revised manuscript.

# Journal Requirements:

**1) Please ensure that the CRediT author contributions listed for every co-author are completed accurately and in full.**
At this stage, the following Authors/Authors require contributions: Elverson Soares de Melo, Sophia Maria Dantas da Silva, Gustavo Barbosa de Lima, Adalucia da Silva, Alexandre Freitas da Silva, Veronica Gomes da Silva, Elisa de Almeida Neves Azevedo, Leticia Welter Rother, Keilla Maria Paz e Silva, Diego Arruda Falcao, Andreza Pamela Vasconcelos, Mayara Matias de Oliveira Marques da Costa, Eduardo Augusto Duque Bezerra, Thiago Franco de Oliveira Carneiro, Erik Matthaus de Lima Paiva, Janaina Correia Oliveira, Matheus Filguera Bezerra, Marcelo Henrique Santos Paiva, Bartolomeu Acioli-Santos, Clarice Neuenschwander Lins de Morais, Tulio de Lima Campos, and Gabriel Luz Wallau.

Please ensure that the full contributions of each author are acknowledged in the "Add/Edit/Remove Authors" section of our submission form.
The list of CRediT author contributions may be found here: <https://journals.plos.org/plosntds/s/authorship#loc-author-contributions>

R – Author Contributions was added after the Acknowledgments section.

2) Please provide an Author Summary. This should appear in your manuscript between the Abstract (if applicable) and the Introduction, and should be 150-200 words long. The aim should be to make your findings accessible to a wide audience that includes both scientists and non-scientists. Sample summaries can be found on our website under Submission Guidelines:
<https://journals.plos.org/plosntds/s/submission-guidelines#loc-parts-of-a-submission>

R – We added the Author Summary.

3) **Please upload all main figures as separate Figure files in .tif** or .eps format. For more information about how to convert and format your figure files please see our guidelines: 
<https://journals.plos.org/plosntds/s/figures>

R – All the figures were converted to the TIFF format.

4) We have noticed that you have uploaded Supporting Information files, but you have not included a list of legends**. Please add a full list of legends for your Supporting Information files after the references list.**

R – We added the legends after the References section.

5) Some material included in your submission may be copyrighted. According to PLOSu2019s copyright policy, authors who use figures or other material (e.g., graphics, clipart, maps) from another author or copyright holder must demonstrate or obtain permission to publish this material under the Creative Commons Attribution 4.0 International (CC BY 4.0) License used by PLOS journals. Please closely review the details of PLOSu2019s copyright requirements here: PLOS Licenses and Copyright. If you need to request permissions from a copyright holder, you may use PLOS's Copyright Content Permission form.
Please respond directly to this email and provide any known details concerning your material's license terms and permissions required for reuse, even if you have not yet obtained copyright permissions or are unsure of your material's copyright compatibility. Once you have responded and addressed all other outstanding technical requirements, you may resubmit your manuscript within Editorial Manager. 
Potential Copyright Issues:

R – We did not use material covered by copyright. All materials of this manuscript were generated by the authors or with no copyright associated and cited accordingly.

i) Figure 2C. Please confirm whether you drew the images / clip-art within the figure panels by hand. If you did not draw the images, please provide (a) a link to the source of the images or icons and their license / terms of use; or (b) written permission from the copyright holder to publish the images or icons under our CC BY 4.0 license. Alternatively, you may replace the images with open source alternatives. See these open source resources you may use to replace images / clip-art:
- [https://commons.wikimedia.org](https://commons.wikimedia.org/)
- <https://openclipart.org/>.

R - The Figure 2C was edited (subsection ordering) by the authors, manually, using Inkscape. No third-party icons, images, or copyrighted materials were used. Therefore, no additional permissions are required for publication under the CC BY 4.0 license.

ii) Figures 1, 2A, 3, 4, 5C, 6, S4, S5, S6, and S7. Please (a) provide a direct link to the base layer of the map (i.e., the country or region border shape) and ensure this is also included in the figure legend; and (b) provide a link to the terms of use / license information for the base layer image or shapefile. We cannot publish proprietary or copyrighted maps (e.g. Google Maps, Mapquest) and the terms of use for your map base layer must be compatible with our CC BY 4.0 license.

Note: **if you created the map in a software program like R or ArcGIS, please locate and indicate the source of the basemap shapefile onto which data has been plotted.**

If your map was obtained from a copyrighted source please amend the figure so that the base map used is from an openly available source. Alternatively, please provide explicit written permission from the copyright holder granting you the right to publish the material under our CC BY 4.0 license.
If you are unsure whether you can use a map or not, please do reach out and we will be able to help you. The following websites are good examples of where you can source open access or public domain maps:
* U.S. Geological Survey (USGS) - All maps are in the public domain. ([http://www.usgs.gov](http://www.usgs.gov/))
* PlaniGlobe - All maps are published under a Creative Commons license so please cite u201cPlaniGlobe, [http://www.planiglobe.com](http://www.planiglobe.com/), CC BY 2.0u201d in the image credit after the caption. (<http://www.planiglobe.com/?lang=enl>)
* Natural Earth - All maps are public domain. (<http://www.naturalearthdata.com/about/terms-of-use/>).

R - We confirm that all maps in Figures 1, 2A, 3, 4, 5C, 6, S5, S6, and S7 were created by the authors in QGIS, and Figure S4 was created in GeoDa. No proprietary basemaps (e.g., Google Maps or MapQuest) were used. The basemap shapefiles onto which all thematic data were plotted correspond to official IBGE geospatial datasets, including territorial boundaries (country, state, and municipality limits), vegetation, climate, and land cover and land use. Only Figure S7 additionally uses data from MapBiomas.

- MapBiomas data are public and available under a Creative Commons Attribution (CC BY) license, as stated in their terms of use:
  [https://brasil.mapbiomas.org/en/termos-de-uso/](https://brasil.mapbiomas.org/en/termos-de-uso/?utm_source=chatgpt.com)
- Information regarding the IBGE license can be found in the official IBGE documentation:
  [https://biblioteca.ibge.gov.br/visualizacao/livros/liv102169.pdf](https://biblioteca.ibge.gov.br/visualizacao/livros/liv102169.pdf?utm_source=chatgpt.com)
  which states (in Portuguese): “O IBGE disponibiliza a MMD e todos os produtos e informações dela derivados sob licença compatível com Creative Commons BY 4.0.”, which can be translated as: IBGE makes MMD and all products and information derived from it available under a license compatible with Creative Commons BY 4.0. This confirms that the “IBGE Malha Municipal Digital” and all derived products are distributed under a license compatible with CC BY 4.0.

**Sources of shapefiles used:**

- **Territorial boundaries (IBGE Malha Municipal Digital):**

<https://www.ibge.gov.br/en/geosciences/territorial-organization/territorial-meshes/2786-np-municipal-mesh/18890-municipal-mesh.html?lang=en-GB>

- **Climate (IBGE):**

<http://geoftp.ibge.gov.br/informacoes_ambientais/climatologia/vetores/brasil/Clima_5000mil.zip>

- **Vegetation (IBGE)**

<https://geoftp.ibge.gov.br/informacoes_ambientais/vegetacao/vetores/escala_250_mil/versao_2023/>

- **Land cover and land use (IBGE):**

<https://www.ibge.gov.br/geociencias/downloads-geociencias.html?caminho=informacoes_ambientais/cobertura_e_uso_da_terra/monitoramento/grade_estatistica/serie_revisada_2022/vetores_compactados/UFs>

- **Land cover and land use (MapBiomas):**

MapBiomas – Collection 2023 of the annual series of Land Cover and Land Use Maps of Brazil, accessed through the official MapBiomas Collection plug-in for QGIS.

Because different figures use different shapefiles, listing all sources in each figure legend would take up too much space. We therefore provided the full set of links in the **Data availability statement** (Lines 679-688).

6) Please amend your detailed Financial Disclosure statement. This is published with the article. It must therefore be completed in full sentences and contain the exact wording you wish to be published.

1) State the initials, alongside each funding source, of each author to receive each grant. For example: "This work was supported by the National Institutes of Health (####### to AM; ###### to CJ) and the National Science Foundation (###### to AM)."
2) State what role the funders took in the study. If the funders had no role in your study, please state: "The funders had no role in study design, data collection and analysis, decision to publish, or preparation of the manuscript."
3) If any authors received a salary from any of your funders, please state which authors and which funders.

R – The Financial Disclosure statement was adjusted.

7) Please revise your current Competing Interest statement to the standard "The authors have declared that no competing interests exist."

R – The Competing Interest statement was adjusted.

# Reviewer #1

Reviewer #1: This is an important and impactful report on OROV surveillance and its current distribution in Brazil. The work is comprehensively presented, methods are sound and the figures are quite informative. Information contained in the manuscript is of seminal importance to the field.

That said, I suggest the authors to address the following concerns prior an eventual publication.

1. The statistical statements seem to contrast with the actual numbers. The authors claim "A correlation analysis between population size and the smoothed incidence rate across municipalities in the Northeast region of Brazil revealed a moderate negative association in the log-transformed space (r = −0.36; p < 2.2×10−16), indicating..." It seems a stretch to draw any conclusion from a Pearson correlation coefficient of 0.36 (positive or negative). Likewise, from a 'weak' autocorrelation.

R - We thank the reviewer for raising this point. Our original analysis was intended only as a descriptive illustration of how population size relates to smoothed incidence rates in log-transformed space, similar to what has been shown in prior OROV studies. Upon this comment, we carefully studied the analytical approach and agree that a Pearson correlation coefficient does not support robust inference in this context. Given the dispersion and the distribution model of the data, we explored more appropriate modeling strategies that support these variations and removed the Pearson correlation analysis from the manuscript.

In the revised Results section, we now report population-stratified summaries of incidence only as descriptive context, mainly to be comparable with results shown in previous studies and to motivate the modeling that follows. Because OROV counts are sparse and many municipalities reported zero cases, we fitted zero-inflated negative binomial (ZINB) regression models to all municipalities (glmmTMB R package, nbinom2 family), using population as an offset to model incidence rates rather than raw case counts. In line with the reviewer's concern, our conclusions now rely on the robust regression results rather than on correlation metrics. Under the ZINB specification, population size was negatively associated with estimated incidence intensity in the count component of the method (β = −0.879, p = 8.4 × 10⁻⁷), suggesting lower estimated incidence intensity in larger municipalities under this specification. We revised the text to reflect this updated analysis (Lines 359-372).

We also described the methods used for this approach in the “Methods” section (Lines 161-173).

2. The authors state that "[...] age distribution, most cases (62.19%) occurred among the 20 to 49 age group." What about the other nearly 40%. **A bar or violin plot of the age distribution would be more informative.**
R - We thank the reviewer for the suggestion. Individual-level age data were not available, as age information was reported exclusively in predefined age groups. Consequently, visualizations requiring individual-level age values, such as violin plots, could not be generated. We therefore add the distribution of cases across all age strata in the paragraph, which represents the maximum level of detail of the available data (Lines 268-272).

3. There is a section in the discussion that is not well connected to the manuscript findings. It is rather structured as proposal pitch and does not contribute to the work. **I suggest to remove entirely or dramatically restructure the following sentences:** "*Mapping areas at risk can guide targeted investment in the continuous training of healthcare professionals for the recognition and clinical management of suspected OROV cases, while also supporting the expansion of access to decentralized molecular testing. Strengthening local diagnostic capacity, including improvements in sample storage and RNA extraction procedures, can minimize viral RNA degradation, improve specimen quality for genomic sequencing, and reduce underreporting. Investment in rapid detection systems is particularly critical given the potential for viral reemergence in municipalities or regions that did not experience major outbreaks but possess climatic and ecological conditions favourable to Culicoides spp. proliferation. In such settings, the low recorded incidence is likely insufficient to establish herd immunity. Lastly, randomized seroepidemiological surveys in affected municipalities are recommended to more accurately estimate prior population-level exposure and, consequently, to assess the risk of future outbreaks based on antibody prevalence*."

R - We revised this section in line with your suggestion, reducing the “proposal” tone and incorporating recommendations from other reviewers (Lines 614-621).

Reviewer #2:

The manuscript entitled “Oropouche Virus Outbreaks in Northeast Brazil between 2024–25 Are Characterized by Sustained Transmission and Spread to Newly Affected Areas” provides a comprehensive characterization of the introduction and spread of OROV in Northeast Brazil during the current outbreak. The study encompasses epidemiological, genomic, and ecological aspects, offering an integrated perspective on the dynamics of OROV transmission in the region.

The manuscript is clear and well written, and the authors provide a detailed description of the methods applied. The methodological approaches are appropriate, and the results are thoroughly and thoughtfully discussed.

The results presented by the authors are highly important for public health, as they provide critical insights into the dynamics of Oropouche virus transmission, support timely detection of outbreaks, and help guide surveillance and control strategies in newly affected regions. I just bring few points to be revised in the manuscript before publication:

1- “Individuals from Pernambuco, Paraíba, Ceará, Maranhão, and Piauí likely acquired the infection while traveling to the Amazonian region (Fig. 2C).”

Please clarify how the authors inferred these likely travel-associated infections in the sentence above, and also the presumed site of infection in Figure 2C. Was this conclusion based on phylogenetic reconstruction, epidemiological information, or another data source? If the inference relied on some epidemiological databases (e.g., SINAN), please reference this use in the Methods section.

**R –** Thank you for pointing this out. This inference was based exclusively on secondary epidemiological data available from the Brazilian Ministry of Health Oropouche Fever Epidemiological Dashboard, which compiles records from the e-SUS Sinan system. These records include information such as the probable place of infection and the individual’s municipality of residence. The presumed site of infection is defined by epidemiological and environmental surveillance teams after evaluating each individual’s travel history and potential exposure, and cases are subsequently recorded as autochthonous or non-autochthonous, together with the geographic location of the presumed site of infection, in the Brazilian notification system (SINAN).

We classified cases as likely travel-associated when the probable place of infection was in a different state from the person’s place of residence. This inference was not based on phylogenetic reconstruction, but solely on epidemiological information recorded in the surveillance system. We add this information to the Methods Section (Lines 145-152).

2- “Additional risk was highlighted in municipalities within the Campina Grande IR (Paraíba)...”
Please, revise this sentence and provide the meaning or context of “IR”.

R – Thank you for raising this point, allowing further clarification. Campina Grande IR” refers to the Campina Grande Intermediate Geographic Region, an official territorial unit defined by IBGE in the 2017 regional division of Brazil, which replaced the previous designations called microregion and macroregion. Intermediate Geographic Regions represent an intermediate scale between the state level and the Immediate Geographic Regions, and they are designed to organize the territory by grouping and articulating Immediate Regions around a higher-order urban pole. This division is shown in Fig. 3A. We revised the manuscript to define the acronym at first use in the methodology section (e.g., “Intermediate Geographic Region, IGR”) (Lines 155-157) and ensure the term is consistently used throughout the text (Lines 379-380).

3- “The findings of this study highlight the urgent need to strengthen surveillance and
response strategies, considering the expansion of Oropouche virus into regions previously
considered non-endemic, but which already face challenges caused by other arboviruses such
as DENV, ZIKV and CHIKV.” Please, include the abbreviations for these three viruses, along their full names, at their first citation in the manuscript.

R – We have now added the full names and abbreviations of these viruses in the first paragraph (Lines 78-81).

# Reviewer #3 (Minor Revision)

PNTD-D-25-01580

It is my honor to review this research article about the Oropouche disease outbreak in Northeast Brazil between 2024-25. The authors provide a comprehensive analysis utilizing the epidemiological and molecular data. Here are my comments:

**Introduction**

In the 4^th^ paragraph of the Introduction, I suggest to update the number of confirmed OROV cases in other American countries using the most recent Pan American Health Organization (PAHO) Epidemiological Update Oropouche in Americas Region on August 13, 2025 (<https://www.paho.org/en/documents/epidemiological-update-oropouche-region-americas-13-august-2025>).

R - We updated the information accordingly (Lines 106-110).

I suggest adding a sentence in the Introduction to briefly introduce the Northeast Region in Brazil. For example, how many states it includes, which states they are, and that is the second most populus region in Brazil. This information would help readers unfamiliar with Brazil better understand the geographic and demographic context of this research. Since there are five paragraphs in the Introduction, the authors can decide the most appropriate location for inserting this information.

R - We added this information at the beginning of the fifth paragraph (Lines 123-126).

**Materials and methods**

Under the section “Segment-Based Phylogenetics and Recombination Filtering,” if Lima et al. (2025) refers to the same paper cited as reference 15, please update the citation to Lima et al. (2024).

R – Thank you for pointing this error. The article was published in the year of 2025; the error is the citation “Lima et al. (2024)”. We corrected it.

**Results**

In the Result section, I noticed that epidemiological week (EW) is the main time unit used to describe confirmed OROV cases. To improve clarity and consistency, I suggest spelling out “epidemiological week (EW)” the first time it is mentioned, and then using only EW in all subsequent references. In addition, I recommend including the actual dates in parentheses. For example, EW 10 in 2024 (March 3-9) instead of using vague terms like “early March,” so that readers have a more precise understanding of the timeline. This suggestion applies to all similar instances throughout the Results section.

R - We implemented these suggestions in the first section of Results.

Under the section “Epidemiological Dynamics of Oropouche Fever in the Northeast of Brazil”, I suggest using “indicating continuous spreading” instead of “indicating an acceleration in the spread of the virus.” While it is true that 11,300 cases were confirmed from January to May 2025, compared with 13,800 confirmed cases in all of 2024, this period corresponds to the wet season when most cases are typically expected. Therefore, there may not be sufficient evidence to conclude an acceleration in virus spread. Thus, I believe describing it as continuous spread is more appropriate.

R – Thank you for this important suggestion. The high number of Oropouche fever cases recorded in the second half of 2024 initially led us to assume that this pattern might persist throughout 2025. However, at the time of this revision, we do not observe the same trend, although data from November and December may still be underestimated due to the time required for laboratory confirmation of cases. Thus, the dynamics observed so far in 2025 appear to differ from those recorded in 2024. We thank the reviewer for the suggestion and have revised the corresponding section of the manuscript (Line 264).

Under the section “Epidemiological Dynamics of Oropouche Fever in the Northeast of Brazil,” the phrase “(~xx% of national cases)” is used to describe the spread of OROV in the Northeast region. I suggest adding a time frame to provide more precise context. For example, “1517 cases of Oropouche fever in 2024 (~11% of 2024 national cases).” The same approach should be applied for 2025: “(~11.4% of 2025 national cases).”

R – We adjusted these sentences (Lines 265-267).

Under the section “Epidemiological Dynamics of Oropouche Fever in the Northeast of Brazil,” the beginnings of the 2^nd^ and 3^rd^ paragraphs provide different information, which may disrupt the flow. In 2^nd^ paragraph: “Autochthonous transmission in Northeast Brazil was first documented in **March**…” In 3^rd^ paragraph: “Although the first autochthonous cases were officially reported in **February** 2024…” Please double check.

R – Thank you for pointing this out. There was a mistake in the 2^nd^ paragraph. We changed from March to February (Line 284).

Under the section “Epidemiological Dynamics of Oropouche Fever in the Northeast of Brazil,” **could the author provide the number of municipalities in Ceará state that reported confirmed cases in 2024 and again in 2025**, particularly where case numbers increased? However, I think this may reflect a continuation of the outbreak from late 2024 into the first half of 2025 as the wet season continuous.

R – Most cases during the 2025 study period in Ceará occurred in Baturité, Aratuba, and Capistrano, municipalities that had already reported high case numbers in 2024. Other municipalities also reported cases in both years, namely Mulungu, Pacoti, and Redenção, but with substantially fewer cases. In contrast, seven additional municipalities reported cases only in 2025: Aracoiaba, Fortaleza, Guaramiranga, Itapiúna, Maracanaú, Quixadá, and Tianguá. We have added this information at the end of the last paragraph of the section “Epidemiological Dynamics of Oropouche Fever in the Northeast of Brazil.” (Lines 327-331)

**Table 1**. I suggest adding a column showing the number of the municipalities in each state as these numbers vary widely: from 75 in Sergipe to 417 in Bahia. Reporting only the percentage does not provide readers with a complete understanding the municipal coverage.

R- We added the information in the Table 1.

Under the section “Municipal Patterns of Oropouche Fever Incidence in Northeastern Brazil”, in the 3^rd^ paragraph, I understand Campina Grande is a municipality in Paraíba but what does “Campina Gande IR” refer to? Cloud this be a typo?

R – Campina Grande IR” refers to the Campina Grande Intermediate Geographic Region, an official territorial unit defined by IBGE in the 2017 regional division of Brazil. Intermediate Geographic Regions represent an intermediate scale between the state level and the Immediate Geographic Regions, and they are designed to organize the territory by grouping and articulating Immediate Regions around a higher-order urban pole. This division is shown in Fig. 3A. We revised the manuscript to define the acronym at first use in the methodology section (e.g., “Intermediate Geographic Region, IGR”) (Lines 155-157) and ensure the term is consistently used throughout the text (Lines 379-380).

**Figure 2**. I may have missed it, but how do the authors distinguish between autochthonous and improved OROV cases?

R – The distinction between autochthonous and imported cases was based on the presumed site of infection, which may differ from the individual’s state of residence. Both variables are available in the Brazilian Ministry of Health surveillance database. The presumed site of infection is defined by epidemiological and environmental surveillance teams, who evaluate each individual’s travel history and potential exposure. Based on this assessment, cases are recorded as autochthonous or non-autochthonous, along with the geographic location of the presumed site of infection in the Brazilian notification system SINAN.

For this study, we had access to information on both the state of residence and the state of the presumed site of infection. Cases in which the presumed site of infection differed from the place of residence were classified as imported. Conversely, cases with coincident locations were classified as autochthonous. We added this explanation to the Figure Legend and to the methods section (Lines 149-152).

**Discussion**

In addition to the fact that “Oropouche fever has historically been underreported in many parts of Brazil and presents symptoms that resemble those other arboviral infections,” another reason is that asymptomatic Oropouche infections are not captured by lab-confirmed cases. **Therefore, one important future study recommendation would be to conduct population-based serological surveys to access the true seroprevalence in a specific location**. While the authors suggest randomized serological surveys, it is not clear how this approach would capture the population most affected. Conduct population-based serosurveys in locations both with and without reported lab-confirmed cases would provide a more comprehensive understanding, allowing estimations of seroprevalence before and after an outbreak and calculation of the attack rate during an outbreak.

R - We thank the reviewer for this important clarification. We agree that randomized serological surveys alone do not necessarily ensure representativeness of the population most affected. Our intention was to recommend population-based serological surveys in affected municipalities as well as in neighboring areas, using probabilistic sampling strategies to estimate population-level seroprevalence, including infections that may not have been clinically detected. The manuscript has been revised accordingly to explicitly reflect this recommendation (Lines 618-621).
